# PTSD symptom clusters associated with short- and long-term adjustment in early diagnosed breast cancer patients

**DOI:** 10.3332/ecancer.2019.917

**Published:** 2019-03-28

**Authors:** Serena Oliveri, Paola Arnaboldi, Silvia Francesca Maria Pizzoli, Flavia Faccio, Alice V Giudice, Claudia Sangalli, Alberto Luini, Gabriella Pravettoni

**Affiliations:** 1 Department of Oncology and Hemato-Oncology, Università degli Studi di Milano, 20122 Milan, Italy; 2 Applied Research Division for Cognitive and Psychological Science, European Institute of Oncology, 20141, Milan, Italy; 3 Data management, European Institute of Oncology, 20141, Milan, Italy; 4 Division of Senology, European Institute of Oncology, 20141, Milan, Italy

**Keywords:** breast cancer, adjustment process, psychological variables, psychological distress

## Abstract

**Objectives:**

We performed an observational prospective cohort study to investigate post-traumatic stress symptoms, emerging after cancer diagnosis, which could influence patients’ short- and long-term adjustment to illness, in order to foster screening measures and management of psychological factors in daily clinical pathways.

**Methods:**

Patients’ post-traumatic stress symptoms, psychological well-being and perceived quality of life were assessed through standardised questionnaires. The Profile of Mood States questionnaire was administered at pre-operative assessment (T0), surgical admission (T1) and discharge from hospital (T2). The Impact of Event Scale and the State-Trait Anxiety Inventory were administered at T0, T1, T2 and 2 years after discharge (T3). At 2-year follow-up, women were also asked to rate their perceived quality of life on a 0–10 visual analogue scale.

**Results:**

Between January 2014 and April 2015, 150 women were enrolled. Results showed that more than 90% of patients experienced post-traumatic stress symptoms after cancer diagnosis (14% with severe symptoms and 76.7% with moderate symptoms) and post-traumatic stress disorder (PTSD) symptoms that persisted up to the 2-year from discharge follow-up, with significant improvement only 2 years after hospital discharge. In particular, mediation models showed that intrusive thoughts impede mood adjustment to the disease during the pre-surgical phase, with anxiety amplifying the negative effect, while symptoms of avoidance are more detrimental in the long term for patients’ quality of life.

**Conclusion:**

PTSD symptom clusters have different influence on short- and long-term reaction to illness. Based on this evidence, appropriate interventions to manage PTSDs in the context of oncology should be developed.

## Background

Multidisciplinary management of breast cancer patients, including psychosocial evaluation and screening, is mandatory since psychological distress in the form of post-traumatic and anxious symptoms is demonstrated to be very frequent from the earliest moments of the illness trajectory and revealed to have an influence on the entire process of adjustment to disease [1].

Breast cancer recently became part of the chronic disease category, and for this reason, the adjustment process does not end with the traditional treatments but encompasses different aspects of medium- and long-term survivorship, such as family planning, coming back to work, social life and the way women plan their future despite the risk of breast cancer recurrence [2].

Studying adjustment has been one of the main purposes of psycho-social oncology since it developed in the early 1970s [3] and this is even more true for breast cancer due to its high prevalence and consistent survival rates [4].

Starting from the diagnosis communication to surgery, studies show that this time often implies a difficult adjustment process in which women have to work through the situation [5]. After surgery, women could feel safer having their cancer removed [1] but complaints and fears emerge later about their cancer treatment. Results in the literature are inconsistent regarding type of surgery and its impact on disease adjustment: in some studies, breast conservative surgery seemed to be correlated with better adjustment as compared to mastectomy; but in other studies, it was associated with poorer quality of life [6, 7].

Studies in the literature suggest a number of psychological correlates of poor adjustment to cancer illness, quality of life and survivorship [8] and these aspects must be detected and assessed from a longitudinal perspective in order to define appropriate care policies, which should integrate the psychosocial point of view. For instance, in a prospective longitudinal study by Lehto *et al* [9] conducted on a consecutive sample of 102 patients evaluated at 3–4 months after breast cancer diagnosis, longer survival was predicted by denying or minimising the fact of having the cancer, while shorter survival was predicted by emotional defensiveness (which refers to non-expression of negative emotions, such as anger and fear) and avoidance towards situations and other people recalling cancer diagnosis. Intrusive thoughts about the disease, trait anxiety, health complaints and problems with sleeping predicted higher levels of distress at follow-up.

Avoidance, repression, passive acceptance and rumination or intrusive thinking predicted a worse psychological adjustment and poorer quality of life even in other studies [10]. With regards to cognitive and perceptual factors, Brandao *et al* [11] review of longitudinal studies showed that intrusion of illness-related thoughts predicted more depressive symptoms.

Only a small number of studies have used mediation analyses to explore how psychological indexes of adjustment to illness might be associated and could affect each other in cancer disease [12, 13].

With this research study, we aimed at enriching literature evidence about the influence of intrusive and avoidant post-traumatic symptoms on mood adjustment to breast cancer and long-term Quality of Life considering a 2-year follow-up. In our exploratory study, we hypothesised that Impact of Event Scale (IES) components intrusions and/or avoidance could have a different influence on mood, emotions and reactions towards the disease in the short term (i.e. a few weeks after diagnosis) and long-term Quality of Life (i.e. up to 2 years after diagnosis) and we tried to detect possible causal relations by means of mediation models.

## Material and methods

### Psychological measures

Study participation was proposed to newly-diagnosed breast cancer patients awaiting mastectomy. It was explained to them that the aim was to monitor their emotional state and their psychological reactions to diagnosis, surgery and discharge from hospital in order to promote better care strategies in these moments of their clinical pathway.

Psychological variables which could affect patient’s mood adjustment to illness were evaluated using the IES [14] and the State–Trait Anxiety Inventory (STAI) in the State form [15] at four points in time: pre-operative assessment T0 (which could occur in a mean time of 33 days after diagnosis), surgical admission (T1), discharge from hospital (T2) and 2 years later (T3).

As it is well known that cancer diagnosis is often experienced like a trauma [16], we decided to administer at all-time points the Impact of Events Scale (IES) [14], one of the most widely used self-report measure within the trauma literature and post-traumatic stress disorder (PTSD) symptoms. The scale is composed of 15 items on a four-point Likert scale (from 0 ‘not at all’ to 4 ‘often’) and it divides traumatic responses into two subscales: Intrusion (7 items), which includes intrusive thoughts, nightmares and intrusive feelings, and Avoidance (8 items), which consists of avoidance of feelings/thoughts and numbness to responsiveness. With the sum of the two subscales, it is possible to compute a total post-traumatic score which can be interpreted with the following dimensions: 0–8 subclinical range, 9–25 mild range, 26–43 moderate range and 44+ severe range. The clinical cut-off point has been set to 26 by the authors who developed the scale.

The STAI is a commonly used measure of trait and state anxiety [15]. It can be used in clinical settings to diagnose anxiety disease and it is also used in research as an indicator of caregiver distress. Its most popular version lists 20 items for assessing trait anxiety and 20 for state anxiety. All items are rated on a four-point Likert scale (e.g. from ‘Almost Never’ to ‘Almost Always’). Higher scores indicate greater anxiety. In the following study, we assessed the state anxiety, excluding trait anxiety, in order to observe emotional reactions in different phases of patient’s clinical pathway (soon after diagnosis, surgical admission, discharge and follow-up).

At pre-operative assessment, surgical admission and discharge from hospital, women were also administered the Italian version of Profile of Mood States (POMS) [17] to evaluate psychological mood adjustment to illness. POMS is a validated psychological test which contains 58 statements that describe people’s feelings. The test requires the patient to indicate for each statement, on a five-point Likert scale ranging from ‘not at all’ to ‘extremely’, how she has been feeling in the past week, including the day of the assessment. It consists of six emotional factors: anger, confusion, depression, fatigue, tension and vigour.

Finally, at the 2-year follow-up, patients were also asked to rate their perceived quality of life on a 0–10 visual analogue scale.

### Patients’ enrolment

Nurses receiving patients in outpatient appointments with the surgeon invited potential participants to meet a research psychologist in charge of presenting the study. After they accepted to participate in the study, a written informed consent was provided and signed by the patient, with personal data and contact details of the research team.

Psychologists contacted the patients and arranged a face-to-face interview for the scheduled day of pre-operative assessment.

Inclusion criteria for patient enrolment were:

- 18 years old or over;
- first cancer diagnosis;
- indication for a mastectomy procedure;
- Italian mother tongue.

Women with a medical history of major psychiatric disorders (patients with diagnosis of full-blown psychiatric disorders diagnosed by *Diagnostic and Statistical Manual of Mental Disorders Fifth Edition* (DSM-5) or International Classification of Diseases-10 (ICD 10), which prevented them to objectively fill out the questionnaires) were excluded so as were women with an indication for pre-surgery neoadjuvant treatment. The study was approved by the Istituto Europeo di Oncologia (IEO) ethical committee and, once enrolled, women were assigned with a progressive number to ensure their anonymity.

At pre-operative assessment (T0), which represented our baseline, women were asked to provide socio-demographic information. Women were also asked to recall the day they received their cancer diagnosis and the tumour dimension description, according to the TNM classification of breast cancer, as described in their medical chart. Secondly, they were asked to complete the questionnaires at baseline.

Psychologists and nurses coordinated themselves in organising, together with patients, further appointments to have participants complete the questionnaires, on the day of admission (T1) and at discharge from the hospital (T2), trying to fit them in with their scheduled medications, check-ups, post-surgery physiotherapy, etc.

Approximately 2 years after hospital discharge (T3), a psychologist phoned all women who had initially agreed to participate in the study and conducted an interview with those who were available. After the fourth failed phone contact, the patient was considered non-respondent and excluded from the follow-up. During the interview, PTSD symptoms and state anxiety were assessed again by means of IES questionnaire, together with perceived quality of life rated on a visual analogue scale.

### Statistical analysis

Descriptive statistics were used to report socio-demographic data, classify the specific kind of tumour and patient’s clinical status. Frequencies and percentages were calculated to assess the number of patients suffering from post-traumatic stress symptoms and anxiety along with the different stages from diagnosis/pre-operative assessment to 2-year follow-up. For continuous variables, mean and standard deviation were calculated. We tested if symptom clusters of PTSD, state anxiety and psychological well-being (POMS) as measures of adjustment towards cancer diagnosis and care, significantly changed at different stages by conducting a repeated measures one-way analysis of variance (ANOVA). Finally, the mediation model was conducted with the PROCESS procedure [18] to test how IES symptom clusters affect POMS total scores and the mediating role of STAI. STAI can be considered a mediator when (i) IES significantly predicts POMS, (ii) IES significantly predicts STAI and (iii) STAI significantly predicts POMS controlling for IES. Sobel test was conducted to compare the strength of the indirect effect of IES on POMS. We performed statistical analysis with the software SPSS version 24.

## Results

Between January 2014 and April 2015, 150 women entered the study and completed both baseline evaluation and T1 follow-up. Fifty-three (35.3%) patients did not complete the questionnaires at T2 and an additional 16 patients (total drop-out of 44%) were not reachable for the 2-year follow-up (T3). Drop-out patients at T2 and T3 did not differ in the investigated aspects when compared to patients who completed all assessment sessions. Failure to complete T2 was mostly due to organisational aspects (such as early hospital discharge and other scheduled medical procedures). Therefore, at T0 and T1, the sample was composed of 150 patients, at T2 of 97 patients and at T3 of 81 patients, all of which completed the questionnaires.

The mean age of the sample was 49 years (SD = 10.9). 30% participants were unmarried, divorced or widowed, while 70% were married or cohabitant; about 74% had children. About 29% participants had an educational level below the secondary school while 71% had completed the high school or were graduated. A total of 26% participants came from the Lombardy region while 74%, a higher percentage, came from outside Lombardy, as observed in our previous studies [19].

Altogether, 83.3% of the sample had a relative or a significant other who had suffered from cancer. Regarding tumour dimension, 44% (*N* = 66) had cancer stage II, 32% (*N* = 48) had cancer stage I, 1.3% (*N* = 2) had cancer stage 0, while for 22.7% (*N* = 34), data were missing. At T0, all patients were not undergoing oncological treatment, while at T3, 9.3% (*N* = 7) had no treatment at that time, 34% (*N* = 28) were undertaking only hormonal therapy and the remaining 56.7% (*N* = 46) had received in the past chemotherapy or radiotherapy and at T3 were receiving hormonal therapy. When women were asked if they had suffered or were suffering from a psychological disturbance, 20% (*N* = 30) answered affirmatively.

At time of pre-operative assessment, 14% (*N* = 21) of the sample presented severe PTSD symptoms while 76.7% (*N* = 115) had moderate PTSD symptoms, while the remaining 9.3% (*N* = 14) did not report symptoms. Before surgery, 11.6% (*N* = 17) manifested severe PTSD symptoms, 71% (*N* = 107) of patients were in the medium clinical range while the remaining 17.4% (*N* = 26) did not manifest symptoms. After surgery, PTSD symptoms were severe in 11.3% (*N* = 11) of patients, medium in 64% (*N* = 62) of women and absent in 24.7% (*N* = 24) of the sample. At 2 years from hospital discharge, only 1.2% (*N* = 1) presented severe symptoms, 66.7% (*N* = 54) showed moderate ones and 32.1% (*N* = 26) did not report PTSD symptoms.

Repeated measures one-way ANOVA on POMS scores determined that mean POMS score differed significantly between time points with an overall improvement of patient’s psychological well-being over time. *Post-hoc* tests using the Tukey’s *post-hoc* (*p* < 0.005) revealed a significant progressive reduction of POMS mean score from T0 to T1 and from T1 to T2. In particular, a significant reduction was observed for all the subscales mean scores from T0 to T2, except for Fatigue-inertia (see Table 1).

Repeated measures one-way ANOVA on STAI-S mean score determined that mean score differed significantly between time points (see Table 1). *Post-hoc* tests using the Tukey’s *post-hoc* revealed a significant increase of STAI-S mean score from T0 (pre-operative assessment) to T1 (surgical admission), and a significant reduction from T1 to T2 (discharge from hospital). From T2 to T3 (2 years after discharge from hospital), another significant increase in anxiety level was observed.

**Figure 1.**
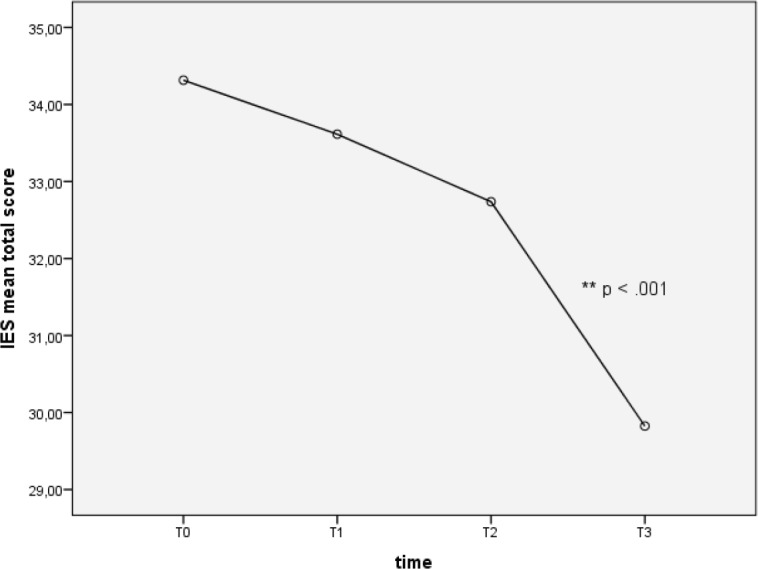
Total PTSD symptoms at different time points.

Repeated measures one-way ANOVA on IES total score showed that post-traumatic symptoms overall decreased between time points (F (2.63, 147.34) = 11.48, *p* < 0.0001). However, *post-hoc* tests (Tukey’s *post-hoc*) revealed a significant difference of IES total mean score exclusively from T2 to T3 (32.74 ± 1.1 versus 29.83 ± 0.83, *p* < 0.05), describing a decrease in severity of post-traumatic symptoms after 2 years from hospital discharge (see Figure 1).

**Figure 2.**
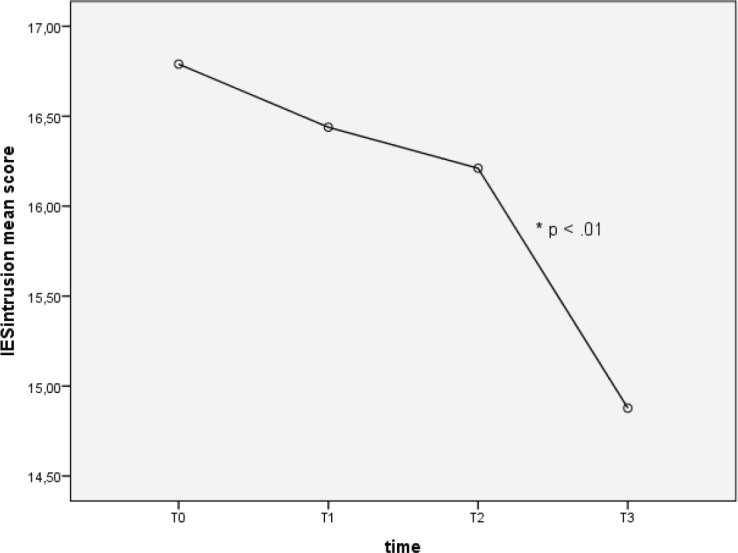
Intrusion scores at different time points.

Repeated measures one-way ANOVA on IES intrusion subscale showed that intrusion overall decreased over time (F (2.41, 134.99) = 4.71, *p* < 0.005). In particular, *post-hoc* tests using the Tukey’s *post-hoc* revealed a significant difference of IES intrusion mean score from T2 to T3 (16.21 ± 0.69 versus 14.88 ± 0.53), *p* < 0.05 (see Figure 2).

**Figure 3.**
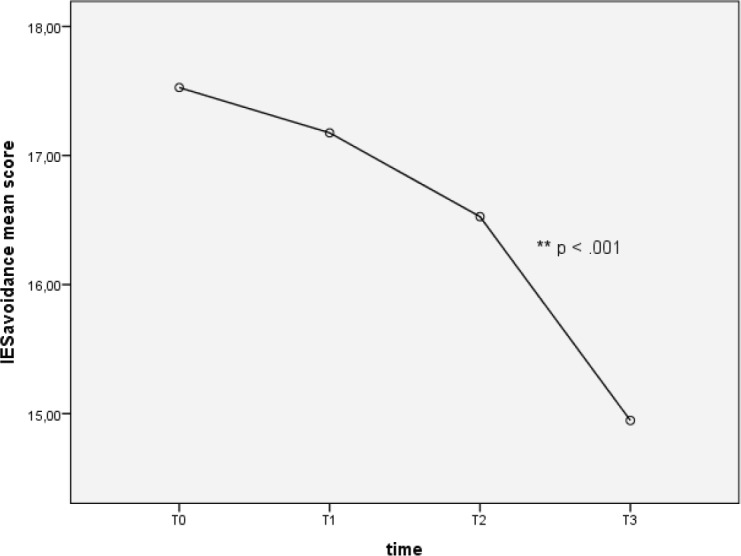
Avoidance at different time points.

Repeated measures one-way ANOVA on IES avoidance score determined that mean score differed significantly between time points (F (2.72, 152.13) = 9.49, *p* < 0.0001). Specifically, Tukey’s *post-hoc* revealed a significant decrease of IES avoidance mean score from T2 to T3 (16.53 ± 0.66 versus 14.95 ± 0.56), *p* < 0.01 (see Figure 3).

**Figure 4.**
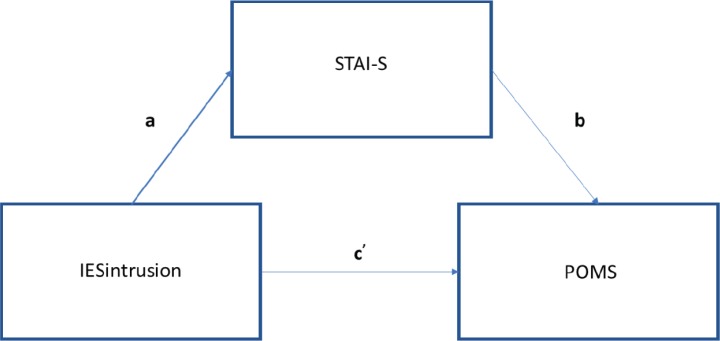
Path diagram of mediation analysis at T1.

Concerning Quality of Life levels stated by patients at 2-year follow-up, mean score on the visual analogue scale (from 0 to 10) was 7.38 (SD 1.96), indicating a good quality of life overall. As the literature has highlighted how PTSD symptoms and anxiety levels are strongly related after breast cancer diagnosis and during the overall clinical pathway [1], we investigated whether and how anxiety levels mediate the relationship between post-traumatic stress (intrusive/avoidant) symptoms and mood adjustment to the disease at different time points. We conducted mediation models at each time point as follows: (1) IES intrusion score predicting POMS score with anxiety as mediator; (2) IES avoidance score predicting POMS score with anxiety as mediator. The regression of IES intrusion subscale and POMS total score at T1 was significant (*b* = 1.76, *t*(108) = 4.8, *p* < 0.001), without considering the anxiety mediator, thus showing that there is a direct effect of post-traumatic intrusive thoughts on mood adjustment to illness. Even the regression of the level of intrusion symptoms on the level of anxiety mediator was significant (*b* = 1.59, *t*(106) = 8.16, *p* < 0.0001), thus demonstrating how PTSD symptoms can cause a considerable increase in anxiety levels. When controlling for the IES, the level of anxiety was significant (*b* = 1.17, *t*(105) = 4.44, *p* < 0.0001). Consistent with full mediation, when controlling for the mediator, the regression model was not significant (*b* = 1.27, *t*(105) = 1.88, *p* = 0.41). The Sobel test found significant full mediation in the model (*z* = 3.88, *p* < 0.0001) (Figure 4). Approximately, 34% of the variance in mood adjustment to illness was accounted by the predictors (*R*^2^ = 0.34).

Differently, there was no full mediation with IES avoidance.

Results indicated that IES avoidance was a significant predictor of anxiety levels at T1 [*b* = 0.634, *t*(105) = 2.76, *p* < 0.005], but the IES avoidance score was a significant predictor of mood adjustment to cancer management after controlling for anxiety levels (*b* = 1.07, *t*(104) = 2.06, *p* < 0.005), consistent with partial mediation.

We conducted mediation analysis with the same predictors at T0 and T2, but regressions did not yield significant results and anxiety did not mediate the relationship between IES subscales scores and POMS scores.

To investigate whether anxiety mediates the relationship between post-traumatic stress symptoms and QoL at T3, we conducted mediation models with IES subscales scores, anxiety levels and QoL. The model that included IES avoidance yielded results consistent with full mediation, while IES intrusion resulted to be a non-significant predictor of QoL (*b* = −0.08, *t*(78) = −1.48, *p* = 0.18).

**Figure 5.**
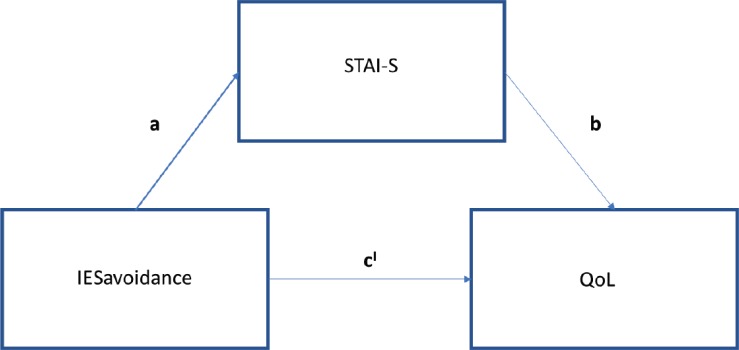
Path analysis IES avoidance at T3.

With regards to IES avoidance, in the first step of the mediation model, the regression of post-traumatic symptoms on QoL, without considering the anxiety mediator, was significant (*b* = −0.1, *t*(78) = −2.57, *p* < 0.05). The regression of the level of avoidance symptoms on the level of anxiety was significant (*b* = 0.75, *t*(78) = 2.74, *p* < 0.05). Consistent with full mediation, when controlling for the mediator, the regression was not significant (*b* = −0.04, *t*(77) = −1.1, *p* = 0.08) (Figure 5). Finally, the Sobel test found full mediation in the model (*z* = −2.5, *p <* 0.05) and approximately, 43% of the variance in QoL was accounted for by the predictors (*R*^2^ = 0.43).

## Discussion

Studying the psychological impact of cancer diagnosis, we have to consider the difference between the normal distress related to cancer diagnosis and therapies and the complete inability in returning to ordinary life after cancer has been diagnosed. In our study, more than 90% of patients experienced post-traumatic stress symptoms after cancer diagnosis (14% with severe symptoms and 76.7% with moderate PTSD). These high percentages of patients presenting PTSD symptoms persisted during surgery and discharge from the hospital, without any significant improvement. This means that breast cancer patients from time of diagnosis communication until hospital discharge relive the moments of check-ups and diagnosis in the form of flashbacks, nightmares, memories or scary thoughts. They also experience discomfort when exposed to factors that recall the disease and the risks associated with it, avoiding all stimuli that may remind them of it.

Previous studies confirmed what observed in our study. Hegel *et al* [20] found that about 10% among newly diagnosed breast cancer patients showed severe PTSD symptoms at the time of their pre-surgical consultation, while Vin-Raviv *et al* [21] observed higher percentages of patients suffering from post-stress disorders (about 23%). In Shelby *et al* [22] study, diagnostic PTSD rates were demonstrated to be low (3%–14%) but PTSD subsyndromal symptomatology occurred in up to 50% of patients. Nevertheless, in the study of Vin-Raviv *et al* [21], symptoms decreased over time, with only 16.5% patients with moderate PTSD symptoms were at 4-months follow-up. Mehnert and Koch [23] showed that a significant decrease of PTSD was observable already in post-surgery phase (only 2.4% patients met the criteria for a mild-to-moderate cancer-related PTSD), especially if treated with breast-conserving surgery. Other studies as well confirmed the long-term improvement [4, 22, 24, 25].

In our study, we found a significant decrease in the amount of patients with PTSD symptoms after 2 years from hospital discharge. Despite this, after such long time, more than half of patients who were asked to recall the very moment of their diagnosis and cancer experience still described their condition in a way that satisfied moderate PTSD diagnosis (about 68% of patients had severe or moderate post-traumatic symptoms after 2 years from hospital discharge). We could explain these data considering that cancer survivors face a sort of ‘sword of Damocles’: cancer survivors, who have a 1 in 5 risk of recurrence [26], feel that their bodies threaten them and have a hard time moving on because they feel that their cancer might come back. Many patients are actively involved in cancer management and care; therefore, they appear strong during treatment as they are fighting cancer. However, when treatments end, although cancer-free, they never really return to their normal self [27–29].

In our study, we observed the progression of state anxiety as an independent parameter. Anxiety is constantly associated with cancer; it is the most prevalent psychological symptom perceived by cancer patients [30] as a response to a threat. In one study conducted by Ashbury *et al* [31], 77% of patients within 2 years of treatment reported anxiety symptoms. Nevertheless, anxiety after cancer diagnosis could be constructive and motivate a patient to deal with her clinical condition or risk: it is not necessarily abnormal [32]. We observed that during the period immediately following diagnosis (pre-operative assessment), general anxiety levels were moderate but they reached a peak close to the surgery phase when patients were admitted to the hospital. At the time of discharge from hospital, the average level of anxiety was significantly reduced compared to the pre-intervention phase; it then increased again after 2 years.

This variation of anxiety levels might demonstrate that anxiety levels change over time. Anxiety that accompanies cancer screenings and test results may be linked to the symbolic idea of loss of ‘immortality’. Instead, there are several factors responsible for anxiety before surgery, such as fear of anaesthesia, concern about lesions that might occur during the procedure, fear of postoperative pain, separation from the family, loss of independence, fear of becoming disabled, not waking up any more, waking up in the middle of the surgery and complications [33, 34]. Meanwhile, anxiety levels years after the cancer disease is linked to worry that cancer will recur.

Despite the presence of PTSD symptoms and anxiety, an overall improvement of patient’s mood adjustment over time was registered, with the exception of fatigue and vigour, because, as said earlier, in this phase, patients are actively engaged in facing the treatments and invest all their mental and physical resources in fighting cancer. Fatigue is perhaps the most common and distressing side effect of breast cancer treatment, with approximately one-third of breast cancer survivors reporting significant levels of fatigue up to 10 years postdiagnosis [35, 36]. At pre-operative assessment, POMS scores were higher, revealing that patients manifested negative feelings. Indeed, we have already seen, cancer diagnosis is like a shock and often triggers feelings of anger, anxiety and fear, depression [37]. The patient has difficulty falling asleep and focusing on something different; moreover, she lives in a state of continuous alert. After diagnosis, the patient might also cope by pretending that nothing is happening [38]. This may not be a conscious decision but an instinctive reaction. The process of denial can be functional to the patient to protect from excessive anxiety and fear of death and gives necessary time to organise the thoughts [38]. It is an adaptive strategy that however becomes weaker with the progression of the disease.

In our study, at surgical admission and at hospital discharge, patients’ mood adjustment to illness improved significantly (decrease in tension, anger, depression and confusion) even if they were about to face a complicated moment of their clinical pathway. This may be due to the shift, after the shock of diagnosis and initial denial, to a phase of bargaining [39]: the patient begins to check what she is able to do and in which aspects she can invest hope. At this stage, a woman regains control over her life and tries her best to cope with the surgical challenge. Vigour and activity significantly decreased as well, since the patient starts to become aware of the losses she is suffering and may experience reactive depression and acceptance [22]. Acceptance means to consider that cancer could be a part of life and the patient stops trying to regain what she has lost and moves on with her life for better or worse.

Since PTSD symptoms and anxiety levels are closely related [1], we observed how PTSD intrusive and/or avoidant symptoms may affect patients’ psychological well-being and mood adjustment to illness through different moments after diagnosis (pre-operative assessment, surgical admission, discharge from hospital and 2 years later) and the way anxiety levels can moderate their impact. Our mediation models showed that at the moment of surgical admission, frequent and intense intrusive thoughts hinder the ability to adjust and cope with the disease, worsening the patient’s mood. Moreover, anxiety levels mediate this relation in a way that the worsening of intrusive thoughts increases anxiety levels, which amplifies the patient’s maladjustment.

In this same phase, symptoms of avoidance also have a negative effect on the mood adjustment to cancer disease, but their impact is not mediated by anxiety levels. This is because avoidant symptoms in some way allow patients to manage anxiety and the fear of surgery [40]. Like denial, this process becomes less and less functional, with worsening of patients’ mood and compliance with the planned therapy. Avoidance shifts the attention, allowing the subject not to focus on the thing that produces anxiety; however, it is psychologically dysfunctional towards an adaptation to a surgical intervention.

Two years after hospital discharge, our data showed that symptoms of avoidance have a significant negative influence on patients’ quality of life, with a small effect of anxiety; when symptoms of avoidance increase, the QoL decreases. Our results were in accordance with trends observed in a previous study by Aguirre-Camacho *et al* [41], who showed that experiential avoidance had negative indirect effect on QoL via depressive symptoms. This means that among PTSD symptoms, avoidance symptoms cluster creates more discomfort at 2-year follow-up in our sample. Avoidance was found to be the strongest predictor of distress and lowered quality of life after cancer in other recent studies [42], and for this reason, interventions focused on reducing cognitive and emotional avoidance should be fostered in this population. In our sample, patients stated to have a good quality of life, having partially overcome disease trauma. We can infer that rather than deny or avoid thoughts concerning the experience of illness, patients who declared to have a good QoL tried to elaborate the positive and negative aspects that have concerned their therapeutic pathway and the way they have dealt with the disease. Not ignoring these thoughts favours a greater adaptation to a new post-illness life arrangement, the acceptance of change and new growth after the illness [43–45]. Instead, patients who prefer to avoid elaborating their experience, because they are subjected to social constraints for instance [40], tend to suffer more 2 years after surgery.

Our data show that women who report being more physically and mentally exhausted and confused at diagnosis, which in turn led to the development of PTSD symptoms, are likely to be exposed to a higher risk of developing long-term difficulties with a potential impact on quality of life and late reaction. Women reporting psycho-physical exhaustion even before surgery should be screened, evaluated and supported by a short-term psychological intervention and undergo subsequent periodic monitoring in order to help them sustain their journey to illness adjustment. This is consistent with new epidemiologic approaches to women’s health [46].

The present study has a number of limitations. We used an old version of the IES [14] where only intrusion and avoidance symptoms scales are present. The more recent version includes the third scale Hyperarousal [47] which was not assessed in our study (we investigated emotional arousal with the STAI) and could be included in future investigations. Moreover, IES was recently considered not to be a very precise measure for PTSD symptoms in cancer patients [48]; however, it remains the most widely tool used in studies on PTSD symptoms after cancer diagnosis and an appropriate measure of intrusion and avoidant symptoms in breast cancer patients [23], Moreover, about 35 and 44 per cent of participants did not complete the T2 and T3 assessment, respectively, and these dropouts limited statistical power of some analysis. Because of dropouts, we were not able to perform the mediation analyses between different time points. We recognise that multiple testing for repeated measures does not allow to draw robust conclusions.

Finally, factors such as the cancer therapies (chemotherapy, hormone therapy or breast reconstruction/mastectomy/lumpectomy) could also play a role in the continuation of anxiety or could also be associated with adjustment to breast cancer in the follow-up period. Further studies should investigate how these variables interact with PTSD symptom clusters and adjustment to the disease.

## Conclusion

Cancer diagnosis can leave a deep wound. It may undermine the feeling of invulnerability and immortality that—whether we aware of it or not—we carry with us. For this reason, it can be considered a traumatic experience. Survivors are not psychologically free: they often perceive suspicious symptoms, are always worried, have flashbacks of the worst periods during illness. Our study found that intrusive thoughts mainly impede mood adjustment to the disease during the treatment period, above all in the pre-surgical phase. Anxiety plays a role in amplifying the effect of intrusive thoughts, sometimes ‘paralysing’ the patient who tries to be compliant with the treatment.

Symptoms of avoidance, on the other hand, are more detrimental in the long term when the most effective process consists in elaborating of this experience rather than attempting to remove the lived experience of what has happened.

In this framework, multidisciplinary teams with mental health professionals should develop appropriate therapeutic pathways to manage traumatic stress disorder in the context of oncology [49].

## Conflicts of interest

The authors declare that they have no conflicts of interest.

## Funding

The authors declare that they had no funding source for this manuscript.

## Figures and Tables

**Table 1.** Mean and standard deviation of the POMS total score, subscales and STAI score.

	T0		T1		T2		
	M	SD	M	SD	M	SD	
POMS total score	67.3	3	61.8	3.2	55.1	3.3	F (1.9, 108) = 16.5*
Tension-anxiety	11.8	5.9	11.2	6.1	9.8	6.7	F (1.8, 171) = 4.6*
Depression-dejection	12.1	10.3	10.5	10.9	8.3	10.2	F (2, 192) = 5.8*
Anger-hostility	11.7	9.8	10.5	11.4	7.5	9.7	F (2, 192) = 10.4*
Vigour-activity	16.1	6.6	15.2	8.2	14	7.5	F (2, 192) = 11.1*
Fatigue-inertia	8	5.4	8	6.3	7.2	5.9	F (2, 192) = 0.8
Confusion-bewilderment	10.2	4.5	9.6	6.5	8	4.9	F (1.9, 181) = 12.3*
STAI	46.2	1.6	49.1	1.6	41.5	1.4	F (2.76, 157) = 11.7**

* *p* < 0.05;

** *p* < 0.01
